# Requirement of Male-Specific Dosage Compensation in *Drosophila* Females—Implications of Early X Chromosome Gene Expression

**DOI:** 10.1371/journal.pgen.1001041

**Published:** 2010-07-29

**Authors:** Natalie Gladstein, Meghan N. McKeon, Jamila I. Horabin

**Affiliations:** Department of Biomedical Sciences, Florida State University, Tallahassee, Florida, United States of America; Max-Planck-Institute of Immunobiology, Germany

## Abstract

Dosage compensation equates between the sexes the gene dose of sex chromosomes that carry substantially different gene content. In *Drosophila*, the single male X chromosome is hypertranscribed by approximately two-fold to effect this correction. The key genes are male lethal and appear not to be required in females, or affect their viability. Here, we show these male lethals do in fact have a role in females, and they participate in the very process which will eventually shut down their function—female determination. We find the male dosage compensation complex is required for upregulating transcription of the sex determination master switch, *Sex-lethal*, an X-linked gene which is specifically activated in females in response to their two X chromosomes. The levels of some X-linked genes are also affected, and some of these genes are used in the process of counting the number of X chromosomes early in development. Our data suggest that before the female state is set, the ground state is male and female X chromosome expression is elevated. Females thus utilize the male dosage compensation process to amplify the signal which determines their fate.

## Author Summary

When substantially different, sex chromosomes present the challenge of not only gene dose inequity between the sexes, in the heterogametic sex where one chromosome (frequently the Y) carries few genes, but also an inequity relative to the autosomes which are diploid. Dosage compensation refers to the process which equates gene dose between the sexes. Recent results, however, indicate that the mammalian X chromosome avoids monosomy and has a level of expression that is two-fold relative to the autosomes. Hyperactive X chromosome expression in *Caenorhabditis elegans* has also been suggested, and dosage compensation in the hermaphrodite appears to lower expression of the X chromosomes to match autosome levels. We find that, before the female state is set in *Drosophila*, the X chromosomes may also express their genes at the two-fold male level and that this level of expression is used to female advantage to consolidate their sex determination. Together, the results suggest that elevated X chromosome expression may be the norm, and that the various dosage compensation processes different organisms utilize reflect a mechanism to counteract an initial hyperactive X chromosome state.

## Introduction

When the sex chromosomes carry substantially different gene numbers, dosage compensation is necessary to equalize gene expression between the two sexes. In the three best studied model systems *Drosophila*, *C. elegans* and mammals where males are XY and females XX, this involves targeting X-specific components which modify the chromatin and transcription of X-linked genes. In each of these cases the end result is different; *Drosophila* upregulates transcription of the male X by about two-fold, *C. elegans* downregulates transcription of both X chromosomes in the hermaphrodite by approximately half, and mammals generally shut down transcription of one of the two female X chromosomes (reviewed in [1]).

As it is the *Drosophila* male which requires dosage compensation, mutation of genes strictly dedicated to this process results in male lethality. The first male specific lethal identified, *maleless* (*mle*; [2]), is indeed involved in dosage compensation as are the next identified male lethals, *msl-1* and *msl-2* [3]. *msl-3* identified by Uchida et al. [4] and *males absent on the first* (*mof*; [5]) complete the proteins collectively known as the *male specific lethals* (*msls*; reviewed in [1], [6], [7]). In addition to these proteins, two RNAs on the X chromosome (the *roX* RNAs), which are not present in females, are also essential for dosage compensation [8]. Although *roX1* and *roX2* show no sequence similarity and do not have an open reading frame that could encode a significantly sized protein, they function redundantly; either *roX* is adequate for function, while loss of both RNAs is required for a failure in dosage compensation and male lethality [9]. The MSL proteins and *roX* RNAs function as a complex, coating the male X chromosome; the X chromosome is hypertranscribed and MOF acetylates histone H4 on lysine 16. Finally, a protein that appears to be part of the dosage compensation complex (DCC) but is required by both sexes is the JIL histone H3 kinase. JIL is also enriched on the male X chromosome but its loss leads to lethality in both sexes [10].

In 1980, Skripsky and Lucchessi [11] reported that females heterozygous for a *Sex-lethal* (*Sxl*) null allele, *Sxl^f1^*, and homozygous for *mle* showed morphological characteristics indicative of sex transformations. *Sxl* is the Drosophila sex determination master switch, which is on in females but off in males. The *Sxl^f1^/+; mle/mle* sex transformation result was confirmed and extended by Uenoyama et al. [12] who observed similar effects with two different *mle* alleles as well as *msl-2* and *msl-3*. This argued that this phenomenon was not unique to *mle*, but likely a general property of the *msls*.

These results, a requirement of male specific genes in females, present a paradox. First, homozygous *msl^−^* females show no sex transformations and are fully viable [3]. Second, besides controlling differentiation, a key function of *Sxl* is to turn *off* the male dosage compensation system to prevent hypertranscription of the two female X chromosomes, which would otherwise lead to female lethality. As a splicing and translation regulator, Sxl alters the splicing and inhibits translation of *msl-2* mRNA so preventing assembly of the DCC [13]–[17]. The absence of MSL-2 also destabilizes MSL-1 and MSL-3 assuring inactivation of the dosage compensation machinery.

The initial activation of *Sxl* is transcriptional, at the *Sxl* ‘establishment’ promoter, *Sxl_Pe_* [18]. In cycle 12 of embryogenesis, *Sxl_Pe_* responds to activating X linked genes (known members: *sisterless-a* (*sis-a*), *sisterless-b* (*sis-b*), *runt* (*run*) and *unpaired* (*upd*)), in conjunction with positive maternal factors such as Daughterless, balancing their dose against the negative effect of genes on the autosomes (*deadpan* (*dpn*), the only identified member) and maternal factors such as Groucho (Gro) and Extramacrochetae (hereafter collectively referred to as the X∶A ratio; reviewed in [19]). Protein from *Sxl_Pe_* transcripts alters the splicing of transcripts from the ‘maintenance’ promoter, *Sxl_Pm_*, first transcribed in cycle 14 in both sexes. In the absence of Sxl protein, default splicing includes a translation terminating exon into the transcripts from *Sxl_Pm_*. As male embryos do not activate *Sxl_Pe_*, Sxl protein is absent and a splicing change on *Sxl_Pm_* transcripts is only effected in females. Females thus set in motion a splicing autoregulatory feedback loop which serves to maintain Sxl expression, and sexual identity, through the rest of the life cycle [20].

Returning to the paradox of a female requirement of male specific genes, one explanation is that XX embryos with only one copy of *Sxl* fail to reliably activate the gene. These XX cells would be male and are presumably eliminated, due to the gene imbalance from inappropriate dosage compensation. However, when one or more of the *msls* is mutant, these masculinized XX cells might survive since assembly of the male DCC is prevented. The resulting clones grow but are sexually transformed, so accounting for the observed sex transformations.

## Results

**Figure 1:**
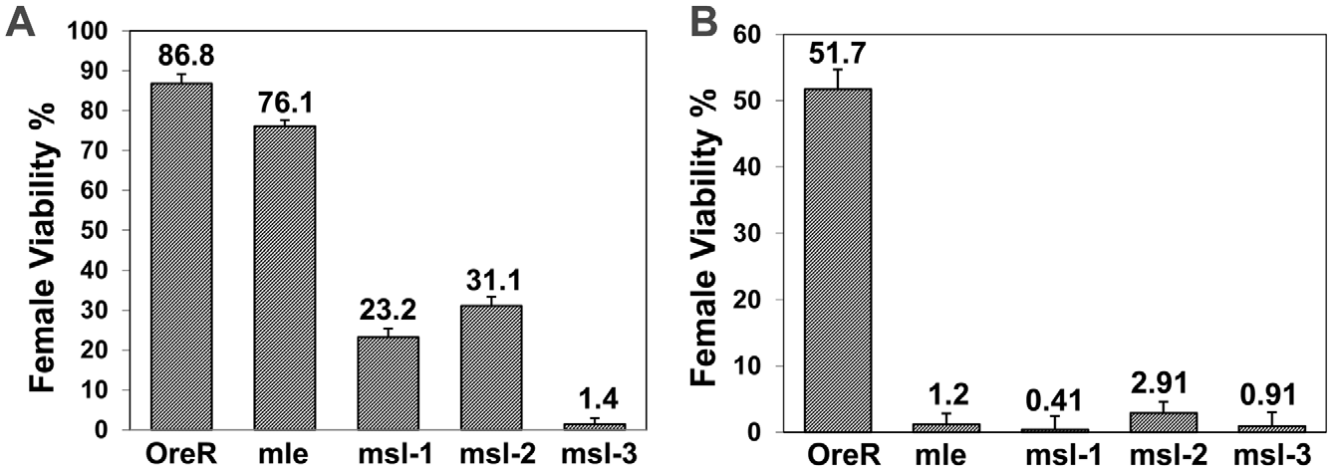
Removal of maternal *msls* reduces female viability when female determining gene dose is compromised. Genotype of mothers shown on x-axis; percent female viability relative to their brothers is shown with percentage standard error. (A) Ore R or homozygous *mle^1^, msl-1^L60^, msl-2^1^ or msl-3^1^* females mated to *y, w, cm, Sxl^fP7B0^*/Y (test classes total n = 439, 1088, 522, 489, 1065, respectively). Females homozygous for the *Sxl^fP7B0^* deficiency are lethal but males with the deficiency are viable. (B) Ore R or homozygous *mle^1^, msl-1^L60^, msl-2^1^* or *msl-3^1^* females mated to *w, sis-b^sc3-1^, sis-a^1^, m*/Y (test classes total n = 270, 934, 593, 849, 555, respectively).

While plausible, the above explanation does not account for the recessive nature of *Sxl* null alleles, which have very high viability (Figure 1A). This high viability requires these females survive the removal of those pockets of male tissue with inappropriate dosage compensation, as *Sxl* hemizygous females show no male differentiation. Figure 1A also shows that the viability of females with only one *Sxl^+^* allele is badly compromised if maternal MSL-3 is removed. Maternal MSL-1 was next most effective followed by MSL-2, while the effect of MLE was negligible relative to wild type. These data demonstrate a synergism of these *msls* with *Sxl* for female viability, as one wild type copy of both *Sxl* and the *msl*, is present in these animals. Contrary to the expectation that female survival might be improved if partially masculinized tissue did not perform male dosage compensation effectively, it would appear that females have a need for the *msls*, when the dose of *Sxl* is halved. Consistent with our findings, some of the *Sxl^f1^/+; msl/msl* combinations in Uenoyama et al. [12] also showed reduced female viability.

### *msls* interact with numerator genes for female viability

The above viability results prompted us to analyze whether key activators of *Sxl* - the numerator genes *sis-a* and *sis-b*, would show a similar interaction with the *msls*. Figure 1B shows that the effect of a *sis-a, sis-b* double mutant chromosome is more extreme than a *Sxl* null, when crossed to mothers mutant for each of the *msls*. The greater effect of the *sis* genes is not surprising, given they function in a dose sensitive process to activate *Sxl* and so determine female sex. What is surprising is that the *msls* interact with the numerators to *promote* female viability.

**Figure 2:**
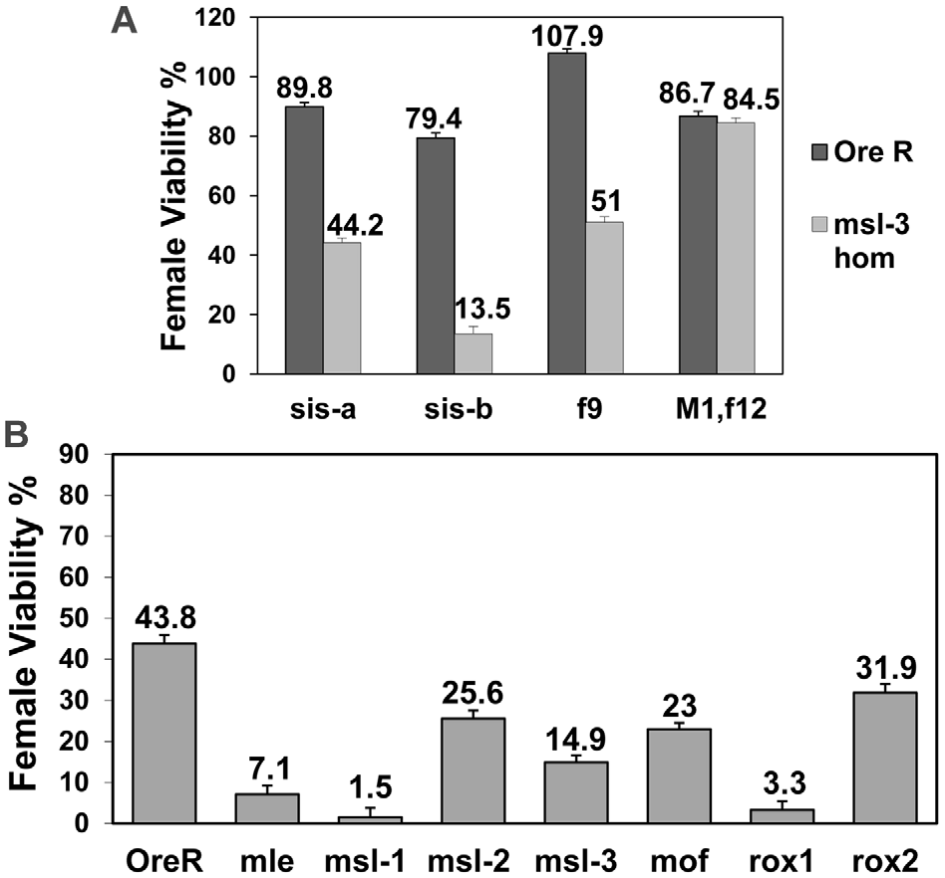
*msls* act early in the female sex determination process, and all key components of the dosage compensation complex, affect female viability. (A) *msl-3^1^* homozygous mothers mated to males mutant for single numerator gene (*sis-a^1^* or *sis-b^sc3-1^*) or *Sxl* early phase (f9) or late phase defective (M1, f12) allele (test class total n = 1135, 1047, 879, 404, 1129, 696, 915, 924, respectively). *sis-b^sc3-1^* cross was done at 29°C, the non-permissive temperature for this temperature-sensitive allele. Percent viability of females relative to their brothers is shown with percentage standard error. Relevant genotype of fathers shown on x-axis. (B) *w, sis-b^sc3-1^, sis-a^1^, m*/FM7 females were mated to either Ore R, *mle^1^*/CyO, *msl-1^L60^*/CyO, *msl-2^1^*/CyO, *msl-3^1^*/TM3, *mof^2^*/Y; 18H1[mof^+^]/+, *roX1^ex6^*/Y or *roX2^−^*/Y males (test class total n = 420, 241, 208, 285, 363, 447, 408, 418, respectively). Percent viability of *sis-a, b*/+ females with the *msl* mutation relative to their FM7/+ sisters also carrying the *msl* mutation is shown, with percentage standard error. For *mof* it is for females that did not receive the 18H1[mof^+^] transgene. Relevant *msl* genotype of fathers shown on x axis.

To test whether the loss of a single numerator gene could also affect females, we performed crosses with reduced dose of either *sis-a* or *sis-b*. Since *msl-3* showed the strongest overall interaction, this *msl* was examined. Figure 2A shows that *sis-a* as well as *sis-b* alone affected females, with *sis-b* having the stronger effect. The *sis* gene interactions suggest that very early steps in the female sex determination process are compromised. Testing two *Sxl* alleles, an early (*Sxl^f9^*) versus a late (*Sxl^M1,f12^*) defective allele, indicated that the early defective allele had an effect, almost as strong as *sis-a* alone, while the late defective allele did not. These data are consistent with the view that early, dose sensitive events in female sex determination are influenced by the *msls*. The late *Sxl* transcripts may not turnover or be as dose sensitive as the early transcripts, so a 50% reduction may not be sufficient to sensitize the females.

*sis-a* and *sis-b* are zygotic in their role in female sex determination. To determine whether the effect observed with the *msls* was maternal or zygotic, reciprocal crosses to Figure 1B were performed. Under these conditions, halving the dose of each of the four *msls*, including *msl-2*, reduced female viability (Figure 2B). The zygotic effect was generally weaker than the maternal.

A maternal effect of *msl-2* is surprising given that the protein is not detected in females [13], [15], [17]. We note that a maternal effect of *msl-2* was also described by Uenoyama et al. [12]. *msl-2* RNA is deposited into the egg (Flybase microarray data; http://www.flybase.org/), so the strength of the zygotic effect is presumably influenced by the amount of maternal protein/RNA of each of the *msls*.

As the *msls*, particularly MSL-3 and MOF, have been shown to bind to both autosomal and X-linked genes where they might perform an unknown role, we wondered whether the entire male DCC, including MOF and the *roX* RNAs, influenced female viability. With the numerator gene dose compromised, halving *mof* dose had an effect, as did *roX1* which was much stronger in effect than *roX2* (Figure 2B). Since the *roX* RNAs function redundantly, the impact of *roX1* and the weaker interaction of *roX2* can be explained by the fact that first expression during embryogenesis is later for *roX2*than for *roX1* [9]. Combined, these results indicate that the *msls* affect an early event and that the entire male DCC is required for promoting female viability.

### Transcription of *Sxl* is affected by the *msls*

The foregoing suggests an event early in *Sxl* expression is altered by the DCC. To directly assess the effect of the DCC on *Sxl* transcription, in situs were performed with *Sxl* probes specific for either the early or late transcripts. Embryos from homozygous mutant *mle^1^*, *msl-1^L-60^*, *msl-2^1^*, *msl-3^1^* or *roX1^ex6^, roX2^−^* double mutant mothers, mated to heterozygous *msl* males were analyzed. For the *roX1, roX2* double mutant embryos, the *roX1^−^, roX2^−^* males have a duplication of *roX2^+^* on their Y chromosome so only the females are *roX1^−^, roX2^−^*.

**Figure 3:**
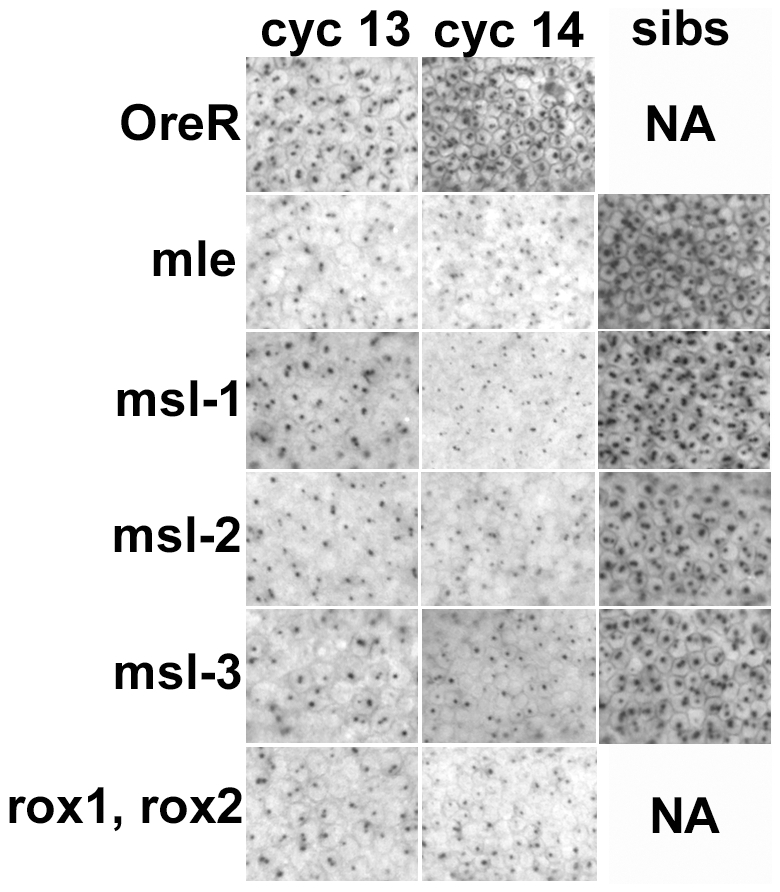
Transcription from the X chromosome dose sensitive promoter of *Sxl*, *Sxl_Pe_*, is reduced in embryos homozygous for the *msls*. In situs on embryos with an early transcript specific probe reveals two dots/nucleus at the sites of transcription on the X chromosomes during cycles 13 and 14 in development. The *roX* double mutants had a reduction in expression in most embryos as all the females are mutant, the *msls* showed reduced expression in about 50% of the embryos (crosses described in text). The ‘sibs’ panels show the signal from normal looking cycle 14 embryos in the same collection, presumably the heterozygous siblings. NA – not applicable. All embryos photographed at 40× mag.

In wild type embryos, *Sxl_Pe_* is not activated until cycle 12, its expression becomes stronger in cycles 13 and 14 before it rapidly ceases expression early in cycle 14. For all the *msls* about half the embryos showed weaker than normal expression of *Sxl_Pe_*, as judged by the size and intensity of the in situ dots on their X chromosomes (Figure 3). The fraction was higher in the *roX1^−^, roX2^−^* cross where all the females are expected to be mutant. These data indicate that the entire DCC complex is used to upregulate transcription from *Sxl_Pe_*.

### A constitutive *Sxl* allele rescues females from *msl*-promoted lethality

**Figure 4:**
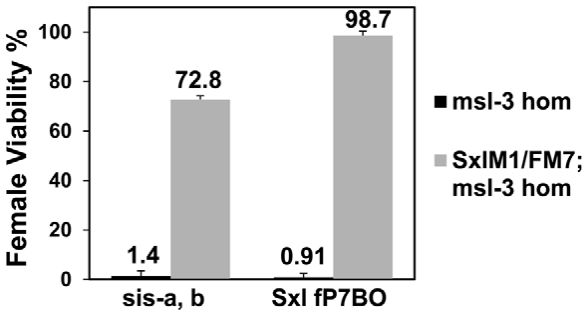
A constitutive *Sxl* allele rescues the female lethality from loss of *msl-3*. *y, cm, Sxl^M1^*/FM7; *msl-3^1^* females mated to *w, sis-b^sc3-1^, sis-a^1^, m*/Y or *y, w,* cm, *Sxl^fP7B0^*/Y males (test class total n = 555, 851, 1065, 681 respectively). Percent viability of females relative to their brothers is shown with percentage standard error. Genotype of fathers shown on x-axis. As the reference males were the balancer FM7/Y, correction for their reduced viability (48.7%) was determined relative to *Sxl^M1^*/+ females from a mating of *y, cm, Sxl^M1^*/FM7; *msl-3^1^* to Ore R males.

If, as the data suggest, the primary reason for female lethality is the failure to activate *Sxl*, a constitutive allele (such as *Sxl^M1^*) which bypasses the X∶A ratio should rescue them. Since *msl-3* showed the strongest interaction in the genetic tests, we determined whether the presence of *Sxl^M1^* could rescue the lethality of *sis-a*, *b* or *Sxl* dose reduction in embryos from mothers homozygous for *msl-3^1^*. The rescue (Figure 4) of 72.8% and 98.7% of the females by *Sxl^M1^* for *sis-a*, *b* or *Sxl* dose reduction, respectively, demonstrates that female lethality is primarily caused by the inadequate expression of *Sxl*.

### Both *Sxl* promoters are affected by the DCC

**Figure 5:**
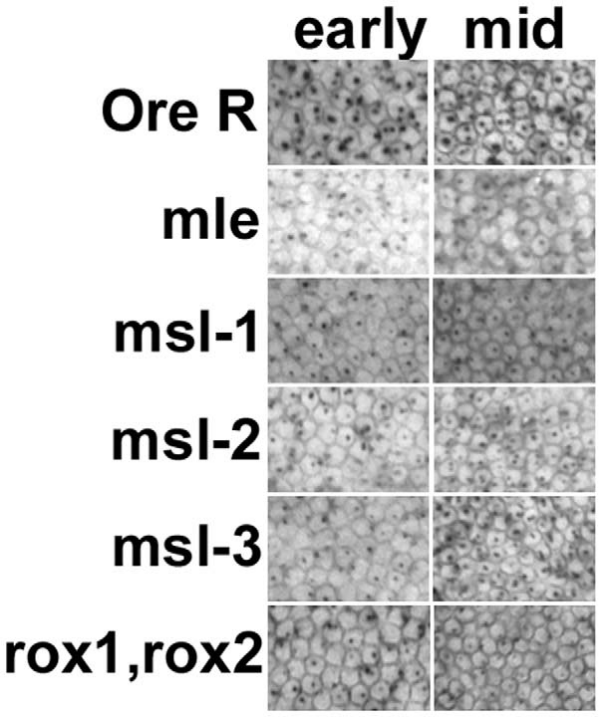
Transcription from the *Sxl* maintenance promoter, *Sxl_Pm_*, is also reduced in embryos homozygous for the *msls*. In situs of embryos using a *Sxl* late transcript specific probe during early and mid cycle 14. As cellularization proceeds during cycle 14, the membranes between the nuclei drop into the embryo, the cell volume changes allowing embryo staging. Only females are shown. Male embryos also transcribe *Sxl_Pm_* – single X chromosome dots – not shown. All embryos photographed at 40× mag.

We next examined whether transcripts from the maintenance promoter, *Sxl_Pm_*, were affected. As shown in Figure 5, this promoter was also affected by loss of DCC components. For *msl-1*, *msl-2* and *msl*-3 about 50% of the embryos, presumably the homozygotes, showed weaker expression. For *mle* and the *roX1^−^, roX2^−^* double mutants almost all the females (2-dots/cell embryos) showed weaker than normal expression. As noted for *Sxl_Pe_*, most of the females are mutant for *roX1^−^, roX2^−^* (excepting the few non-disjunction embryos that also receive the Y with a duplicated *roX2^+^* gene), however, only 50% of the embryos are homozygous for *mle^1^*. The *mle^1^* data suggest the maternal contribution of MLE is important for *Sxl_Pm_* expression, an effect that appears distinct from the loss of the DCC since for *Sxl_Pe_* only half the embryos were affected. This may be an outcome of *Sxl_Pm_* relying more heavily on maternal MLE compared to the other *msls*. Alternatively, because MLE also affects the stability of *roX1* RNA, which has a larger role in sex determination than *roX2* (Figure 2B), the effect of mutating MLE may be amplified as it not only eliminates the maternal MLE but also reduces the levels of *roX1* RNA, acting as a double mutation. Despite this unexplained effect on *Sxl_Pm_* by maternal MLE, the data together indicate that both *Sxl* promoters are susceptible to the DCC and suggest that *Sxl*, which resides on the X, is a dosage compensated gene. Consistent with the idea that transcription elongation and not initiation is altered by the DCC [21], transcription from both *Sxl* promoters, which are regulated by different factors, is affected.

**Figure 6:**
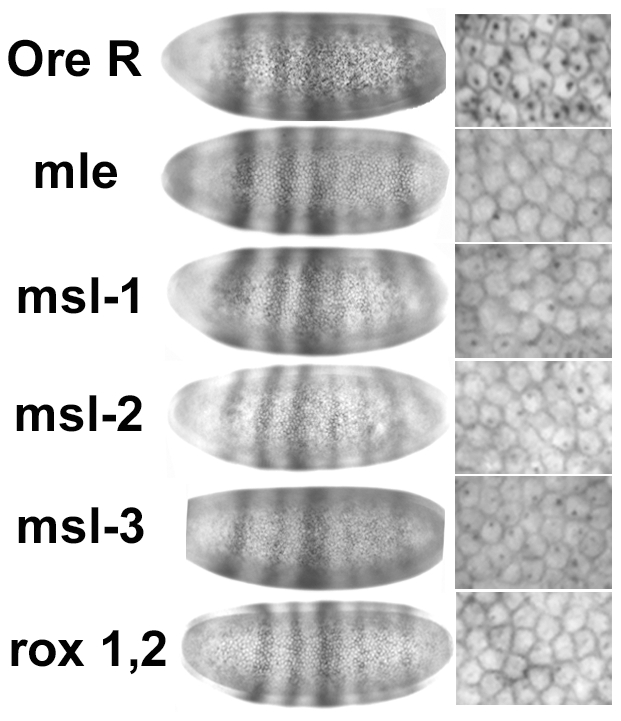
Reduced in situ signal in *Sxl* is not accompanied by reduced signal in another unrelated gene. In situs for *Sxl_Pm_* transcripts performed simultaneously with the segmentation gene *hairy*. Embryos from *msl* mothers show the normal *hairy* striping pattern of expression for the same developmental stage as Ore R embryos (left set of panels), but poor *Sxl_Pm_* signal (right set of panels—enlarged view to show cells). All embryos photographed at 40× mag.

Although the in situs of Ore R embryos did not show the distinctly different classes we observed with the *msl* embryos, to control for the possibility that the quality of the in situs was responsible for generating a poor signal in half the embryos from *msl* mutant mothers, in situs for *Sxl_Pm_* transcripts were simultaneously performed with a distinguishable second probe - the segmentation gene *hairy* which has a striped pattern of expression. As seen in Figure 6, embryos from *msl* mutant mothers that are at the same developmental stage as Ore R embryos have comparable *hairy* stripes but poor *Sxl_Pm_* signal, indicating that the poor signal is not an artifact of the in situs but an effect of the *msls* on *Sxl* transcription.

### Quantitation of *Sxl* expression in embryos from *msl* mothers

The in situs are qualitative and the nuclear dots detect transcription directly off the chromosomes, indicating only high levels of transcription. For a better measure, we performed quantitative RT-PCR analysis on 2–3 h and 2.5–3.5 h embryos for *Sxl_Pe_* and *Sxl_Pm_* expression, respectively. Embryos were from homozygous mutant mothers for *msl-2^1^*, *msl-3^1^* or *roX1^ex6^, roX2^−^* double mutants, mated to heterozygous *msl* males. As for the in situs, in the *roX1^−^, roX2^−^* embryos only the females are doubly mutant as males have a duplication of *roX2^+^* on their Y. RNA levels were normalized to tubulin levels and compared to Ore R embryos which were set to 1.

**Figure 7:**
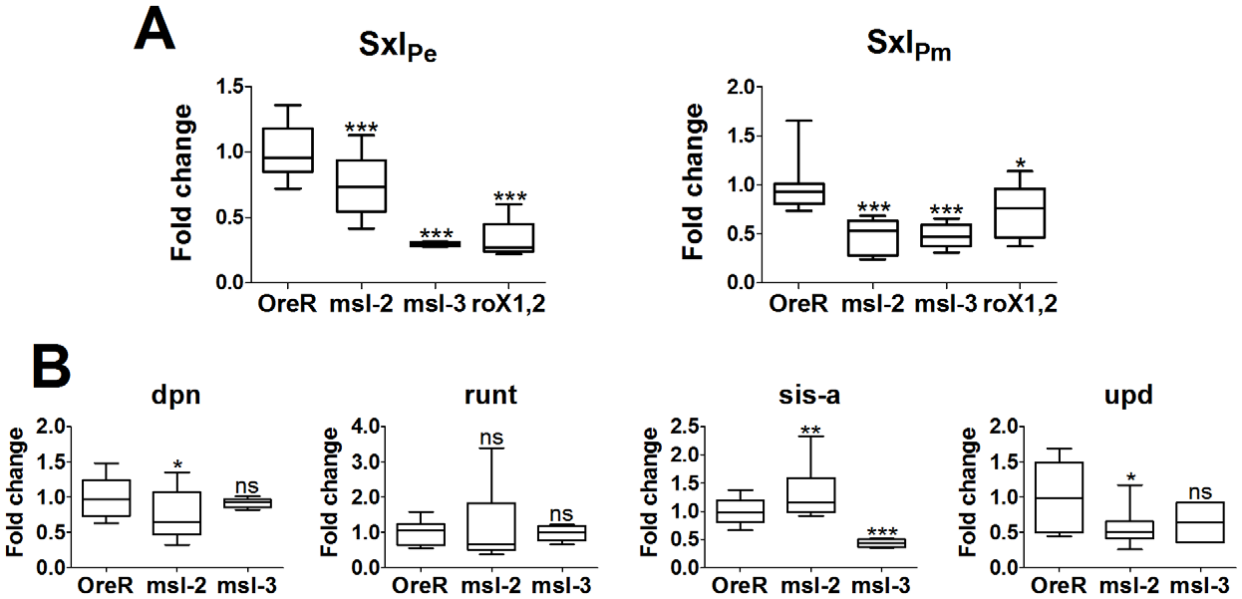
Change in mRNA expression for dosage compensated and control genes compared to Ore R. All replicates are plotted and display the 25 and 75 percentiles (boxed), median (line in boxes), max and min (whiskers) of the data set. Significance is measured using an unpaired t-test with a Welch's variance correction, levels indicated above whiskers as _***_ for p<.001, _**_ for .001<p<.01, _*_ for .01<p<.05, and ‘ns’ for p>.05. (A) *Sxl_Pe_* and *Sxl_Pm_* transcripts show a highly significant drop in expression when DCC function is compromised by mutation in *msl-2, msl-3* or the *roX* genes. Embryo genotype on the x-axis. As noted in the text, the lower significance in the change in *Sxl_Pm_* levels for the *roX-* genotype is most likely the influence of males which have a wild type *roX2* gene. (B) *dpn* and *run*, show almost no significant change in expression, with the slight exception of *dpn* in the *msl-2^1^* mutants. *sis-a* and *upd* show significant change in a DCC-compromised backgrounds, with specific differences between mutant genotypes. *sis-a* shows the expected drop in expression for *msl-3^1^*embryos but shows a slight increase in expression for *msl-2^1^*embryos. This appears to arise from an additional role of MSL-2, affecting mRNA levels (see Figure S1). *upd* is affected by the loss of MSL-2 but is essentially unaffected by the loss of MSL-3, as would be expected for a gene with close DCC entry sites.

Figure 7 shows that *Sxl_Pe_* is expressed at lower levels than Ore R embryos in all three *msl* genotypes. The median for *msl-2^1^* embryos was slightly above, for *msl-3^1^* and *roX1^−^, roX2^−^* embryos the median slightly below half of Ore R. The medians for *Sxl_Pm_* were also close to half, except for the *roX1^−^, roX2^−^* genotype which was closer to 0.7. *Sxl_Pm_* is transcribed in both sexes and all the males have a functional *roX2* gene in the *roX1^−^, roX2^−^* embryos. In these embryos, *Sxl_Pm_* gave a value of 0.7, suggesting males are transcribing *Sxl_Pm_* at close to normal levels while the females express *Sxl_Pm_* at close to half. This would suggest that functional *roX2* RNA is present mid-way through cycle 14, a little earlier than in situs can detect [9].

A value close to 0.5 for both promoters (excepting *Sxl_Pm_* for *roX1^−^, roX2^−^*) was a little surprising given the in situ results which show about half the embryos have close to normal levels of transcription. It suggests that the DCC may be upregulating the expression of *Sxl* by a little more than two-fold, not unlike the *roX* genes [22]. Alternatively, and not mutually exclusive, it may also indicate that at 2–3 h of development most of the DCC is assembled primarily from maternal reserves and the presence of one wild type chromosome in half the embryos (from the heterozygous fathers) makes a small contribution. With respect to *Sxl_Pe_*, the qRT-PCRs score embryos whose average age is slightly younger than the in situs, at cycles 13 and 14 (2.75–3.25 h). Close examination of those in situs shows few embryos in early cycle 13 with uniform, wild type levels of *Sxl_Pe_* expression. However, when the membranes begin to drop between the nuclei later in cycle 13, the class with more uniform expression resembling wild type, is more readily observed (data not shown). By late cycle 13 and cycle 14, the zygotic contribution of the wild type chromosome from the heterozygous fathers must begin, and the two different classes are more readily apparent in cycle 14 embryos (Figure 3). For *Sxl_Pm_*, the data are more consistent with the DCC having slightly greater than a two-fold effect.

Effects of the DCC were also scored for some of the sex determination genes in the 2–3 h collections from homozygous *msl-2^1^* or *msl-3^1^* mothers. *msl-3^1^* embryos show *sis-a*, like *Sxl*, with a median expression close to 0.5, while *run* and *dpn* gave medians close to 1 as expected for non-dosage compensated genes (*sis-b* could not be reliably scored as it has an anti-sense transcript, CG32816). *upd* appeared reduced to ∼0.7 but this was not statistically significant and the data showed greater variability than for the other genes. This may be because *upd* begins expression later (cyc 13; [23]), and half the embryos are beginning to perform normal dosage compensation. Also, there are 2 DCC high-affinity sites (see Discussion below) relatively close to *upd*. These are predicted to make *upd* less sensitive to the loss of MSL-3, since MSL-3 is required for spreading of the DCC from its initial entry sites.

For *msl-2^1^* embryos, the *upd* median did drop to ∼0.5, consistent with the loss of MSL-2 having a greater effect than MSL-3 for genes with close DCC entry sites. *run* did not show a significant change from wild type. However, unlike for the *msl-3^1^* embryos, *sis-a* was slightly elevated relative to wild type, while *dpn* mRNA, at a low level of significance, showed a small decrease. As the *msl-3^1^* embryos show that *sis-a* is dependent on the DCC, these latter data suggest that besides dosage compensation, MSL-2 may have an additional role, one that perhaps affects mRNA stability. MSL-2 affects the steady state levels of the *roX* RNAs [24]; such an activity could explain the greater variability in the values we measured for *msl-2^1^* embryos. To test this, in situs of s*is-a* mRNA were performed to determine if over time, the mRNA levels would show a change consistent with accumulation. Indeed, we found this to be the case (Figure S1), suggesting that in the case of *sis-a* MSL-2 may serve to destabilize its RNA. During the early cycles, embryos from *msl-2^1^* mothers had signal which was generally weaker than wild type, but by cycle 12 when the message has its highest accumulation in wild type [25], the accumulated levels in the *msl-2^1^* embryos were even higher. While alternative explanations, e.g. repression of the *sis-a* promoter by MSL-2 are also plausible, this effect would have to occur at some but not all stages of *sis-a* transcription and be independent of the DCC, as loss of MSL-3 shows the predicted 2-fold drop in *sis-a* mRNA levels.

Despite the suggestion of an additional role beyond dosage compensation for MSL-2, the qRT-PCR data show that the 2 *Sxl* promoters are expressed at approximately half their normal levels by the loss of the DCC. Expression of other X-linked genes also appears to be similarly affected, very clearly evident in the *msl-3^1^* embryos. This indicates the DCC functions relatively early, and may also affect the handful of genes known to be expressed during these early stages of embryonic development [26], [27].

### Transient expression of the DCC in females

**Figure 8:**
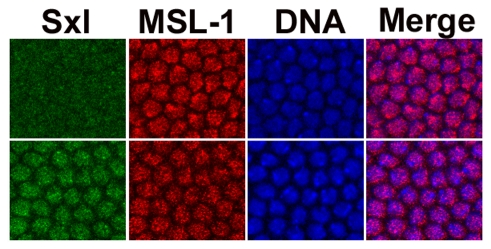
Sxl and the DCC are transiently co-expressed in female embryos. During mid cycle 14, Sxl (green) and the DCC (red - mostly from anti-MSL-1 antibody) can be simultaneously detected in females. MSL-1 overlaid on DNA (blue) signal (Merge). Top set of images from males, as determined by the lack of specific Sxl signal, lower set is of females. The ant-MSL-1 signal in the female nuclei is less intense and generally covers a larger area than in males, as might be expected for their double dose of X chromosomes. As previously noted [46], the signal in males is more frequently at the nuclear periphery.

The data argue for a role of the male DCC in females, a function not ascribed to it, and the complex has not been detected in female embryos [28]–[30]. Our data suggest that prior to the full activation of *Sxl* there is a brief window of male dosage compensation in females, after which Sxl protein is predicted to shut down MSL-2 expression, and destabilize the entire DCC. Not all anti-MSL antibodies have been reported to detect the complex at this early stage, even in males (see [30]). Given this limitation, we used an anti-MSL-1 antibody from the Lucchesi lab which has high sensitivity and enhanced the signal with an M3TAP construct [31]. These embryos were co-stained with anti-Sxl antibodies and closely examined around the cellular blastoderm stage. Figure 8 shows that there is indeed a very brief stage, in mid cycle 14, when it is possible to simultaneously detect both Sxl and the DCC in females. The ant-MSL-1 signal in the female nuclei is not as bright and generally covers an area of DNA larger than in males, presumably the two X chromosomes.

## Discussion

The effect of the DCC on *Sxl* transcription early in embryogenesis explains the contradiction of why genes that are normally off in females are required to promote their viability. In the absence of the *msls* and a functional male DCC, transcription of some of the genes on the X chromosome as well as *Sxl* is not elevated by two-fold. This effectively weakens the X∶A ratio and lowers the levels of the *Sxl* early as well as late transcripts, which when low enough leads to female lethality. With respect to *Sxl_Pe_*, insufficient levels of early protein are produced and splicing of *Sxl_Pm_* transcripts into the female mode is compromised. With respect to *Sxl_Pm_*, a reduction may compromise establishment of the female state as the autoregulatory splicing feedback loop would have to rely on reduced mRNA and protein levels.

In the absence of mutations in feminizing genes, lowering of *Sxl* expression by the *msls* is not detrimental, presumably as the very process which would lead to female lethality - male dosage compensation - is no longer functional, while the Sxl positive autoregulatory feedback loop slowly establishes itself into the female state. Without the DCC, however, reduced dose of feminizing genes, particularly the dose sensing X-linked genes or numerators, lowers *Sxl_Pe_* transcript levels further, and has deleterious consequences for females. *Sxl* dose also has an effect, but unlike the numerators *Sxl* is not strictly dose sensitive, and not unexpectedly, when its copy number is halved, has a less profound effect on female viability. It should be noted that extremely low levels of Sxl in females, even in the absence of the male DCC is lethal [32]. Sxl protein directly performs a female dosage compensation role, reducing the levels of X-linked genes such as *run* [33]; the latter is not upregulated by the male DCC [[34]; Figure 7].

### Early X chromosome expression is elevated in females

Our data indicate that some of the earliest expressed genes on the X, the numerators as well as *Sxl*, are dosage compensated. Dosage compensation is a chromosome wide phenomenon, and, at the least, the effect of the *msls* can be detected as early as cycle 13. Previous work timed the DCC in males at cellular blastoderm (Stage 5, [30]) and early gastrulation (Stage 6, [29]). Our data (qRT-PCRs and in situs) suggest dosage compensation sets in earlier, by 2–3 h in development and appears to initially rely on maternal stores and the zygotic expression of the *roX* RNAs (*roX1* primarily). As discussed by McDowell et al. [30] antibody sensitivity sets the limit for the prior studies. The present studies relied on different assays, which may account for the difference. Indeed, we were also unable to detect convincing signal in males, which is normally stronger, much before blastoderm by antibody staining (Figure 8). It is also possible that early in development the DCC is harder to detect directly as there are fewer genes being transcribed, so less of the complex may have assembled onto the X chromosome before cycle 14, when the mid-blastula transition occurs and there is a large transcriptional burst. The zygotic expression of *roX1* RNA has been placed at around 2 h of embryogenesis [9], consistent with the effects we observe.

Targeting of the DCC to the X chromosome, rather than the autosomes, is thought to rely on transcription marks, sequence elements (∼150 MREs – MSL recognition elements and ∼130 HAS – high affinity sites), and other unknown elements [35], [36]. The identified sequence element set is still incomplete since the two data sets show an overlap of 69%; it is predicted that the X chromosome may have as many as 240–300 elements (reviewed in [7]). Examination of the published MRE and HAS shows the closest element to *Sxl* ∼139 Kbp 5′ of the gene. This distance is on the large side, although it should be noted that all elements which target the DCC to genes on the X remain to be identified; as an example, the *white* gene has its closest known MRE/HAS 93 Kbp away but its mini form in transgenes, which does not include this site, is clearly dosage compensated. Finally, ChIP data (modENCODE, Flybase) show *Sxl* with strong H4K16 acetylation marks, a modification dependent on the DCC. ChIP data for JIL-1 kinase also suggest the DCC is at *Sxl*.

None of the other sex determination genes, other than *upd* (two 3′ elements at ∼5.6 and 6.8 Kbp away) had an element within 10 Kbp (*sis-a* ∼26 Kbp, *sis-b* ∼38 Kbp), consistent with the observation that the *msls* involved in spreading the DCC from its entry sites on the X (MSL-3, MOF and the *roX* RNAs), are required for their elevated expression. *upd*, the exception, showed greater sensitivity to the loss of MSL-2 than MSL-3, as might be expected for dosage compensation which is less dependent on spreading. An interesting correlation is that *run* which is not compensated, had its closest elements ∼343 (5′) and ∼273 Kbp (3′) away, further than the rest of the other known key sex determination genes.

### Default mode is male

**Figure 9:**
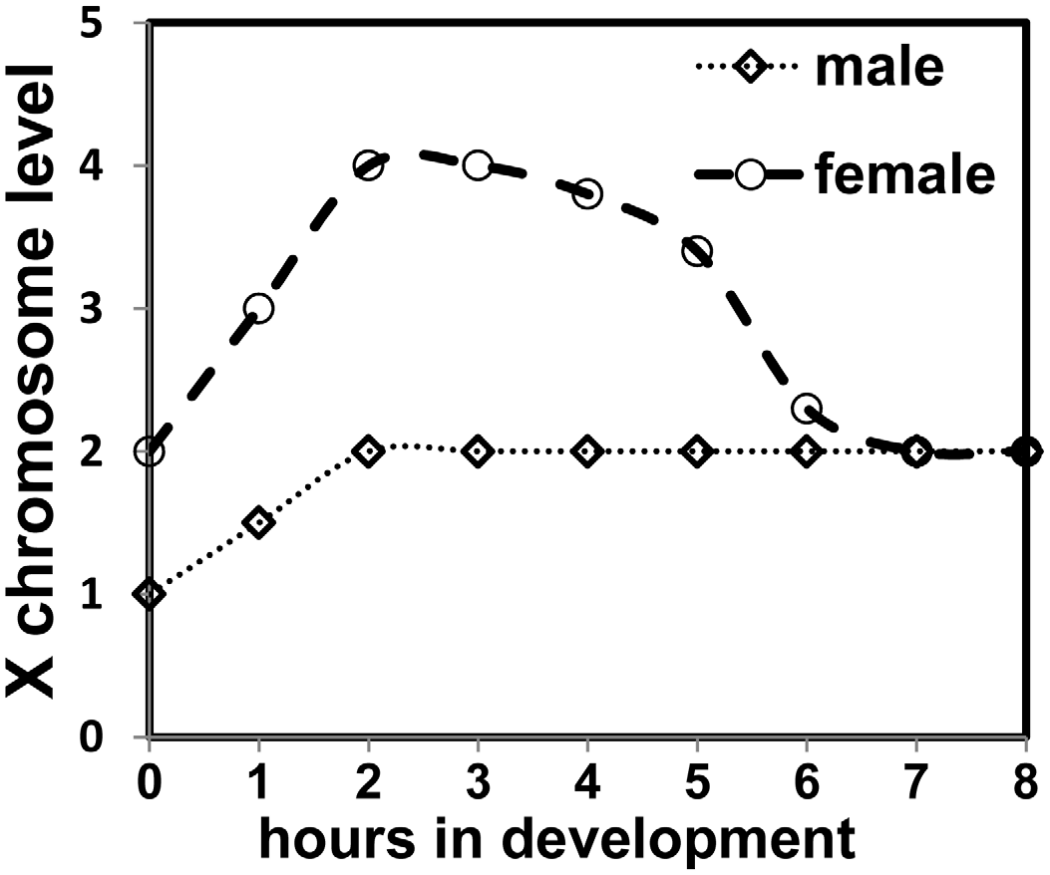
Model depicting X chromosome expression levels in the two sexes. At 25°C cycle 13 is around 2 h 15 min and cycle 14 concludes around 3 h 15 min. Reduction of the DCC effect is shown as being gradual as the female mode of *Sxl* splicing is established, and repression of MSL-2 expression is more complete.

By using the DCC before the female state is established, *Sxl* capitalizes on the default male state. Transcription from *Sxl_Pe_* is amplified, an effect unique to females as males do not transcribe from *Sxl_Pe_*. Determination of female identity is thus consolidated. As expression of Sxl protein levels is established, Sxl protein subsequently shuts down the DCC and eliminates the very difference in gene dose between the sexes which set in motion, as well as augmented, its own activation. Implicit, is that before the establishment of Sxl expression, each X-chromosome in females is transcribed at 2X levels, as in males, and our qRT-PCR data of some of the key sex determination genes would support this view. The conventional X∶ A ratio would then be 4∶2 rather than 2∶2, and in males 2∶2 rather than 1∶2 (Figure 9).

In that there is a 2-fold difference between the sexes, this scenario is mathematically the same. However, there are practical and functional implications. An X∶ A ratio that is transiently 4∶2 rather than 2∶2 in females, would have some of the X-linked genes which activate *Sxl_Pe_* at twice the level of their putative counteracting autosomal or denominator genes. In a screen which seeks suppression of a female-specific lethal condition due to a decrease in numerator elements, it would require the equivalent of two autosomal genes to be mutated to reestablish an X∶ A ratio favorable for female survival. Obtaining two mutations in genes functioning in the same process at once is unlikely, which may have skewed the outcome of screens which sought to identify zygotic autosomal genes. It may not be a coincidence that the only autosomal acting component identified is *dpn* [37], [38]. As both Dpn and Run bind the co-repressor Gro [39], [40] but have opposing effects on *Sxl_Pe_*, it has been speculated they may antagonize each other [39], [41]. Screens may have repeatedly identified *dpn* as it would be counteracting a gene expressed at its chromosomal equivalent, since *run* is not upregulated by the male DCC.

### Elevated X chromosome transcription

On a more general level, our data suggest an upregulation of transcription of the Drosophila X, and may reflect a universal requirement of elevated X chromosome expression to avoid monosomy. Recent microarray analysis of mouse ES cells indicates that mammalian dosage compensation is more complex than previously thought: there is higher expression of the X chromosome relative to the autosomes giving them equivalence, i.e. chromosome per chromosome the X is overexpressed by about two-fold relative to each autosome [42]–[44]. As differentiation proceeds, females lose expression of one of their X chromosomes, silencing it through inactivation. Put in other words, the mammalian X chromosome is not monosomic in expression but rather is hyperactive, and the process of dosage compensation appears to shut down elevated X chromosome transcription in females. (Hyperactive X chromosome expression in *C. elegans* has also been suggested [42], so dosage compensation in the hermaphrodite would then serve to lower the X chromosomes to match autosome levels).

In this regard, Drosophila would not be very different from mammals except that rather than inactivating one of the female X's, Sxl inactivates the mechanism which upregulates X chromosome specific expression. In all cases, dosage compensation avoids tetrasomy of the X. What the components are which specifically upregulate the mammalian or *C. elegans* X chromosome—the Drosophila male DCC counterpart—remain to be determined.

## Materials and Methods

### Fly crosses

Flies were reared under uncrowded conditions on standard cornmeal medium. All crosses were done at 25°C; Ore R was the wild type control. Progeny were counted out to 8 days from the first day of eclosion. Description of genes can be found in Flybase (http://www.flybase.org/).

### In situs and immunofluorescence

These were done as in Erickson and Cline [25]. The *Sxl* early (407 nt) and late (1039 nt) transcript specific probes were generated by the primers, respectively: 5′ GTTCCACTCGTGACAAGTCC 3′and 5′ GTTTCTAAGCAGATCCCG 3′; 5′ GCGAAACGTGCACACTGC 3′ and 5′ GGGCGATGCTTGCATGTTGC 3′ (T7 promoter sequence removed). For *hairy*, the primers 5′ CCAGAACCTGCTGCTCAT TCG 3′ and 5′ GGGAAAGCGGCTA ACCTCGTTC 3′; for *sis-a* the primers 5′ CAAAATGCACTACGCCGACG 3′ and 5′ GCATCGTGTCCAACATGACG 3′ were used. All in situs were repeated at least once. Each batch was done simultaneously with an Ore R control, and had sufficient embryos so that several representatives of each cycle could be examined. M3TAP embryos [31] were stained for Sxl (mouse) and MSL-1 (rabbit) as previously described [45]. To enhance the MSL signal, the M3TAP was first bound (blocked) by the same anti-rabbit fluorescent secondary used for the anti-MSL-1 primary before addition of the primary antibody.

### qRT–PCRs

Embryos were collected on apple juice agar plates for one hour and aged for the appropriate time. They were washed off the plate, dechorionated with 50% chlorox, washed extensively with 1x PBST and frozen at −80°C. RNA was extracted from the frozen embryos using tri-reagent as per manufacturer's protocol. An additional phenol extraction was performed on the purified RNA, followed by DNAse treatment. A PCR test was performed on the RNA to confirm the lack of DNA, after which 4 ug of the RNA was reverse transcribed (RT) with AMV RT at 50°C for 15 min followed by 1.5 h at 42°C. A small amount (2 ng) of *Sxl* primer (5′ CGT GTC CAG CTG ATC GTC GG 3′) was added to the oligo-dT mix (100 ng) per RT, as the stage specific 5′ exons of *Sxl* are distant from the polyA tail. The quantitative PCRs were performed in triplicate on a Bio-Rad iQ5 thermocycler; C_t_ values that showed a difference of greater than 0.5 from the other two replicates were discarded. For each genotype a minimum of 3 separate RNA samples was analyzed. PCR products were between 200 and 300 bp; primers for *Sxl_Pe_* 5′ CTGTTCGACCATGTCGTCCTA C and CTA CCACCGCTGCCCAGCGAC, *Sxl_Pm_* 5′ GTGGTTATCCCCCATATGGC 3′ and 5′ CTA CCACCGCTGCCCAGCGAC 3′, *sis-a* 5′ CGTATACGCACCGTATCGCGG 3′ and 5′ GCATCGTGTCCAACATGACG, *runt* 5′ CGACGAAAACTACTGCGGCG 3′ and CCAGCCAAGCGGGATTCAGC, *upd* 5′ GAAAGCGGAACAGCAACTGG 3′ and 5′CAGGAACTTGTAGTTGTGCG 3′, *dpn* 5′ CCGATTATGGAGAAACGTCGC 3′ and 5′ CTGAGCCGCTGACGAACACC. Statistical data analysis was completed using Microsoft Excel and GraphPad Prism.

## Supporting Information

Figure S1 MSL-2 affects accumulation of *sis-a* mRNA. In situs of embryos using a *sis-a* probe shows early expression (cycles 9 and 10) to be slightly lower in embryos from homozygous *msl-2^1^* mothers. By cycle 12, however, the levels accumulated in embryos from *msl-2^1^* mothers were higher than wild type.
(1.00 MB TIF)
