# Supplementary figures and images for: Requirement of Male-Specific Dosage Compensation in Drosophila Females—Implications of Early X Chromosome Gene Expression
Source: PLoS Genet. 2010 Jul 29;6(7):e1001041. doi: 10.1371/journal.pgen.1001041 (PMC2912388; doi:10.1371/journal.pgen.1001041)

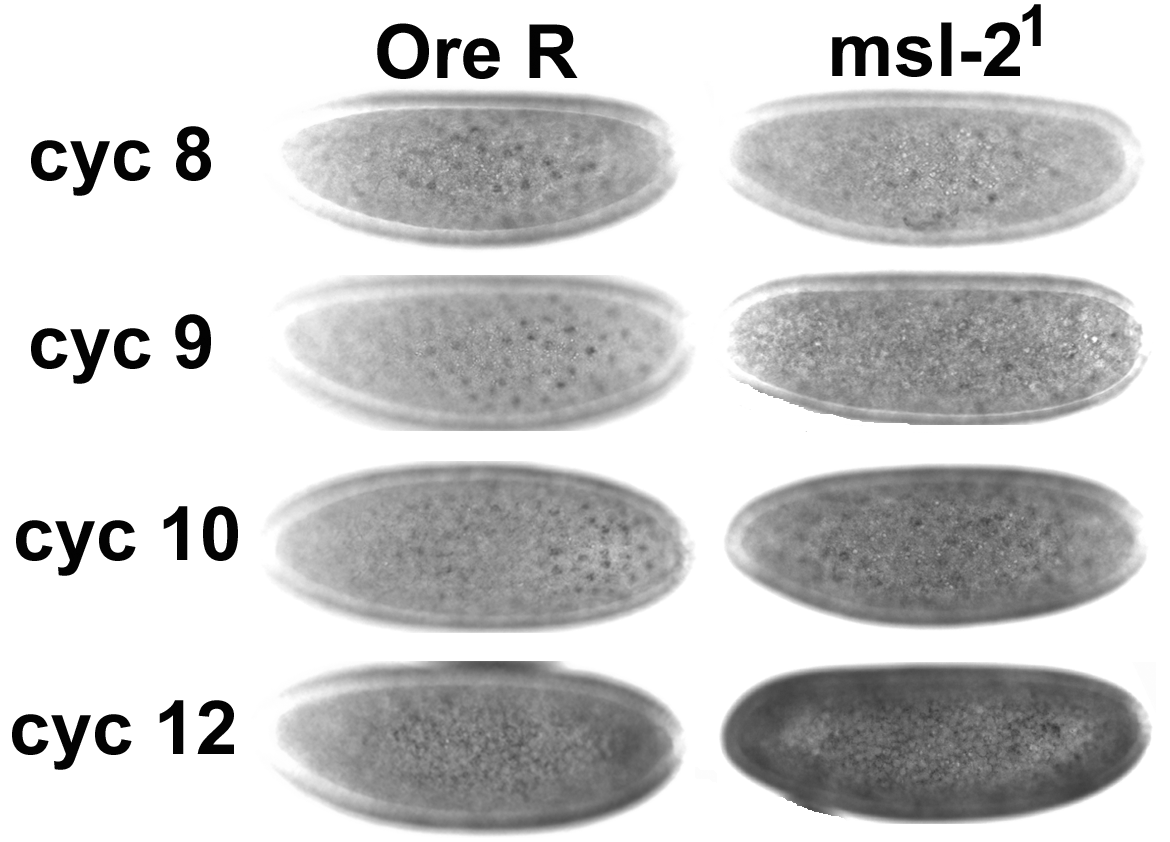

Supplement: Figure S1 — MSL-2 affects accumulation of sis-a mRNA. In situs of embryos using a sis-a probe shows early expression (cycles 9 and 10) to be slightly lower in embryos from homozygous msl-21 mothers. By cycle 12, however, the levels accumulated in embryos from msl-21 mothers were higher than wild type. (1.00 MB TIF) [file pgen.1001041.s001.tif]
